# Supplementary material for: Optimizing road safety: integrated analysis of motorized vehicle using lattice ordered complex linear diophantine fuzzy soft set
Source: PeerJ Comput Sci. 2024 Jul 23;10:e2165. doi: 10.7717/peerj-cs.2165 (PMC11323125; doi:10.7717/peerj-cs.2165)
Supplement: Supplemental Information 1 [file peerj-cs-10-2165-s001.pdf]

Supplementary

In this section based on the data as reported in $(A_M, \widehat{H})$, $(A_I, \widehat{H})$, and $(A_D, \widehat{H})$, we determine the steps of an algorithm as follows.

Step 1: The $\widehat{LOCLDFSM}$ is formulated from above mentioned $\widehat{LOCLDFSS}$.

$$M = \begin{bmatrix} [\langle(0.6,0.7),(0.3,0.2)\rangle,\langle(0.7,0.7),(0.3,0.3)\rangle] & [\langle(0.7,0.8),(0.2,0.2)\rangle,\langle(0.7,0.8),(0.3,0.2)\rangle] & [\langle(0.8,0.9),(0.2,0.1)\rangle,\langle(0.8,0.8),(0.1,0.1)\rangle] \\ [\langle(0.8,0.7),(0.2,0.2)\rangle,\langle(0.7,0.8),(0.3,0.2)\rangle] & [\langle(0.8,0.8),(0.1,0.2)\rangle,\langle(0.8,0.8),(0.1,0.2)\rangle] & [\langle(0.9,0.9),(0.1,0.1)\rangle,\langle(0.9,0.8),(0.1,0.1)\rangle] \\ [\langle(0.6,0.9),(0.2,0.1)\rangle,\langle(0.7,0.8),(0.3,0.1)\rangle] & [\langle(0.8,0.9),(0.1,0.1)\rangle,\langle(0.8,0.9),(0.2,0.1)\rangle] & [\langle(0.9,0.9),(0.1,0.1)\rangle,\langle(0.8,0.9),(0.2,0.1)\rangle] \\ [\langle(0.7,0.6),(0.3,0.3)\rangle,\langle(0.7,0.8),(0.3,0.2)\rangle] & [\langle(0.8,0.7),(0.1,0.3)\rangle,\langle(0.8,0.8),(0.2,0.2)\rangle] & [\langle(0.9,0.8),(0.1,0.2)\rangle,\langle(0.8,0.8),(0.2,0.2)\rangle] \end{bmatrix}$$

$$I = \begin{bmatrix} [\langle(0.5,0.6),(0.4,0.3)\rangle,\langle(0.6,0.6),(0.4,0.4)\rangle] & [\langle(0.6,0.7),(0.3,0.3)\rangle,\langle(0.6,0.7),(0.4,0.3)\rangle] & [\langle(0.7,0.8),(0.3,0.2)\rangle,\langle(0.7,0.7),(0.2,0.2)\rangle] \\ [\langle(0.7,0.6),(0.3,0.3)\rangle,\langle(0.6,0.7),(0.4,0.3)\rangle] & [\langle(0.8,0.7),(0.2,0.2)\rangle,\langle(0.7,0.7),(0.2,0.3)\rangle] & [\langle(0.8,0.8),(0.2,0.1)\rangle,\langle(0.9,0.8),(0.1,0.1)\rangle] \\ [\langle(0.7,0.8),(0.3,0.2)\rangle,\langle(0.6,0.7),(0.4,0.2)\rangle] & [\langle(0.8,0.8),(0.2,0.2)\rangle,\langle(0.7,0.8),(0.3,0.2)\rangle] & [\langle(0.9,0.9),(0.1,0.1)\rangle,\langle(0.8,0.8),(0.2,0.2)\rangle] \\ [\langle(0.6,0.5),(0.4,0.4)\rangle,\langle(0.6,0.8),(0.4,0.2)\rangle] & [\langle(0.7,0.6),(0.2,0.4)\rangle,\langle(0.7,0.8),(0.3,0.2)\rangle] & [\langle(0.9,0.7),(0.1,0.3)\rangle,\langle(0.8,0.9),(0.2,0.1)\rangle] \end{bmatrix}$$

$$D = \begin{bmatrix} [\langle(0.8,0.6),(0.2,0.4)\rangle,\langle(0.6,0.5),(0.4,0.4)\rangle] & [\langle(0.8,0.6),(0.2,0.4)\rangle,\langle(0.6,0.6),(0.4,0.4)\rangle] & [\langle(0.8,0.7),(0.2,0.3)\rangle,\langle(0.6,0.6),(0.3,0.3)\rangle] \\ [\langle(0.6,0.5),(0.3,0.4)\rangle,\langle(0.6,0.6),(0.3,0.4)\rangle] & [\langle(0.7,0.6),(0.3,0.3)\rangle,\langle(0.6,0.6),(0.3,0.4)\rangle] & [\langle(0.9,0.7),(0.1,0.3)\rangle,\langle(0.8,0.7),(0.2,0.2)\rangle] \\ [\langle(0.6,0.7),(0.4,0.3)\rangle,\langle(0.5,0.6),(0.4,0.3)\rangle] & [\langle(0.7,0.7),(0.3,0.3)\rangle,\langle(0.6,0.7),(0.4,0.3)\rangle] & [\langle(0.8,0.8),(0.2,0.2)\rangle,\langle(0.7,0.7),(0.3,0.3)\rangle] \\ [\langle(0.8,0.7),(0.2,0.3)\rangle,\langle(0.5,0.7),(0.4,0.3)\rangle] & [\langle(0.8,0.7),(0.2,0.3)\rangle,\langle(0.6,0.7),(0.4,0.3)\rangle] & [\langle(0.9,0.8),(0.1,0.2)\rangle,\langle(0.7,0.8),(0.3,0.2)\rangle] \end{bmatrix}$$

Step 2: Estimate the Score Matrix $\widehat{S}(M)$, $\widehat{S}(I)$ and $\widehat{S}(D)$.

$$\widehat{S}(M) = \begin{bmatrix} \begin{array}{c} \frac{1}{4}((0.6-0.3)+(0.7-0.2) \\ +(0.7-0.3)+(0.7-0.3)) = 0.4 \end{array} & \begin{array}{c} \frac{1}{4}((0.7-0.2)+(0.8-0.2) \\ +(0.7-0.3)+(0.8-0.2)) = 0.525 \end{array} & \begin{array}{c} \frac{1}{4}((0.8-0.2)+(0.9-0.1) \\ +(0.8-0.1)+(0.8-0.1)) = 0.7 \end{array} \\ \begin{array}{c} \frac{1}{4}((0.8-0.2)+(0.7-0.2) \\ +(0.7-0.3)+(0.8-0.2)) = 0.525 \end{array} & \begin{array}{c} \frac{1}{4}((0.8-0.1)+(0.8-0.2) \\ +(0.8-0.1)+(0.8-0.2)) = 0.65 \end{array} & \begin{array}{c} \frac{1}{4}((0.9-0.1)+(0.9-0.1) \\ +(0.9-0.1)+(0.8-0.1)) = 0.775 \end{array} \\ \begin{array}{c} \frac{1}{4}((0.6-0.2)+(0.9-0.1) \\ +(0.7-0.3)+(0.8-0.1)) = 0.575 \end{array} & \begin{array}{c} \frac{1}{4}((0.8-0.1)+(0.9-0.1) \\ +(0.8-0.2)+(0.9-0.1)) = 0.725 \end{array} & \begin{array}{c} \frac{1}{4}((0.9-0.1)+(0.9-0.1) \\ +(0.8-0.2)+(0.9-0.1)) = 0.75 \end{array} \\ \begin{array}{c} \frac{1}{4}((0.7-0.3)+(0.6-0.3) \\ +(0.7-0.3)+(0.8-0.2)) = 0.425 \end{array} & \begin{array}{c} \frac{1}{4}((0.8-0.1)+(0.7-0.3) \\ +(0.8-0.2)+(0.8-0.2)) = 0.575 \end{array} & \begin{array}{c} \frac{1}{4}((0.9-0.1)+(0.8-0.2) \\ +(0.8-0.2)+(0.8-0.2)) = 0.65 \end{array} \end{bmatrix}$$

$$\widehat{S}(I) = \begin{bmatrix} \begin{array}{c} \frac{1}{4}((0.5-0.4)+(0.6-0.3) \\ +(0.6-0.4)+(0.6-0.4)) = 0.2 \end{array} & \begin{array}{c} \frac{1}{4}((0.6-0.3)+(0.7-0.3) \\ +(0.6-0.4)+(0.7-0.3)) = 0.325 \end{array} & \begin{array}{c} \frac{1}{4}((0.7-0.3)+(0.8-0.2) \\ +(0.7-0.2)+(0.7-0.2)) = 0.5 \end{array} \\ \begin{array}{c} \frac{1}{4}((0.7-0.3)+(0.6-0.3) \\ +(0.6-0.4)+(0.7-0.3)) = 0.325 \end{array} & \begin{array}{c} \frac{1}{4}((0.8-0.2)+(0.7-0.2) \\ +(0.7-0.2)+(0.7-0.3)) = 0.5 \end{array} & \begin{array}{c} \frac{1}{4}((0.8-0.2)+(0.8-0.1) \\ +(0.9-0.1)+(0.8-0.1)) = 0.7 \end{array} \\ \begin{array}{c} \frac{1}{4}((0.7-0.3)+(0.8-0.2) \\ +(0.6-0.4)+(0.7-0.2)) = 0.425 \end{array} & \begin{array}{c} \frac{1}{4}((0.8-0.2)+(0.8-0.2) \\ +(0.7-0.3)+(0.8-0.2)) = 0.55 \end{array} & \begin{array}{c} \frac{1}{4}((0.9-0.1)+(0.9-0.1) \\ +(0.8-0.2)+(0.8-0.2)) = 0.7 \end{array} \\ \begin{array}{c} \frac{1}{4}((0.6-0.4)+(0.5-0.4) \\ +(0.6-0.4)+(0.8-0.2)) = 0.275 \end{array} & \begin{array}{c} \frac{1}{4}((0.7-0.2)+(0.6-0.4) \\ +(0.7-0.3)+(0.8-0.2)) = 0.425 \end{array} & \begin{array}{c} \frac{1}{4}((0.9-0.1)+(0.7-0.3) \\ +(0.8-0.2)+(0.9-0.1)) = 0.65 \end{array} \end{bmatrix}$$

$$\widehat{S}(D) = \begin{bmatrix} \frac{1}{4}((0.8-0.2)+(0.6-0.4) & \frac{1}{4}((0.8-0.2)+(0.6-0.4) & \frac{1}{4}((0.8-0.2)+(0.7-0.3) \\ +(0.6-0.4)+(0.5-0.4))=0.275 & +(0.6-0.4)+(0.6-0.4))=0.3 & +(0.6-0.3)+(0.6-0.3))=0.4 \\ \frac{1}{4}((0.6-0.3)+(0.5-0.4) & \frac{1}{4}((0.7-0.3)+(0.6-0.3) & \frac{1}{4}((0.9-0.1)+(0.7-0.3) \\ +(0.6-0.3)+(0.6-0.4))=0.225 & +(0.6-0.3)+(0.6-0.4))=0.3 & +(0.8-0.2)+(0.7-0.2))=0.575 \\ \frac{1}{4}((0.6-0.4)+(0.7-0.3) & \frac{1}{4}((0.7-0.3)+(0.7-0.3) & \frac{1}{4}((0.8-0.2)+(0.8-0.2) \\ +(0.5-0.4)+(0.6-0.3))=0.25 & +(0.6-0.4)+(0.7-0.3))=0.35 & +(0.7-0.3)+(0.7-0.3))=0.5 \\ \frac{1}{4}((0.8-0.2)+(0.7-0.3) & \frac{1}{4}((0.8-0.2)+(0.7-0.3) & \frac{1}{4}((0.9-0.1)+(0.8-0.2) \\ +(0.5-0.4)+(0.7-0.3))=0.375 & +(0.6-0.4)+(0.7-0.3))=0.4 & +(0.7-0.3)+(0.8-0.2))=0.6 \end{bmatrix}$$

Step 3: Determine the Utility Matrix as below.

$$\widehat{U}(M,I,D) = \begin{bmatrix} 0.275-(0.4-0.2)=0.075 & 0.3-(0.525-0.325)=0.1 & 0.4-(0.7-0.5)=0.2 \\ 0.225-(0.525-0.325)=0.025 & 0.3-(0.65-0.5)=0.15 & 0.575-(0.775-0.7)=0.5 \\ 0.25-(0.575-0.425)=0.1 & 0.35-(0.725-0.55)=0.175 & 0.5-(0.75-0.7)=0.45 \\ 0.375-(0.425-0.275)=0.225 & 0.4-(0.575-0.425)=0.25 & 0.6-(0.65-0.65)=0.6 \end{bmatrix}$$

Step 4: Evaluation of the Total Score Matrix.

$$Total\ Score\ Matrix = \begin{bmatrix} 0.075+0.1+0.2=0.375 \\ 0.025+0.15+0.5=0.675 \\ 0.1+0.175+0.45=0.725 \\ 0.225+0.25+0.6=1.075 \end{bmatrix}$$

Step 5: Ranking of the alternatives is $l_4 > l_3 > l_2 > l_1$
