# Supplementary material for: Optimizing road safety: integrated analysis of motorized vehicle using lattice ordered complex linear diophantine fuzzy soft set
Source: PeerJ Comput Sci. 2024 Jul 23;10:e2165. doi: 10.7717/peerj-cs.2165 (PMC11323125; doi:10.7717/peerj-cs.2165)
Supplement: Supplemental Information 5 — The authors have not employed any coding-centric software in the algorithmic design. Despite this, we have articulated methodological steps in a general template below. These steps can also be implemented through machine learning tools after translating into executable code. Statement of the Problem: Road accidents are frequently happening in India due to collisions. There is no guarantee for safer driving. Adaptive Cruise Control (ACC) fills this gap and enables a convenient and safer driving experience by monitoring other vehicles and objects on the road with the use of sensors. It also helps the driver keep a steady vehicle speed. [file peerj-cs-10-2165-s005.docx]

**Code**

**Manuscript Title: A Systematic Analysis of Motorized Vehicles using Lattice Ordered Complex Linear Diophantine Fuzzy Soft Set**

In the aforementioned manuscript, the authors have not employed any coding-centric software in the algorithmic design. Despite this, we have articulated methodological steps in a general template below. These steps can also be implemented through machine learning tools after translating into executable code.

**Statement of the Problem**: Road accidents are frequently happening in India due to collisions. There is no guarantee for safer driving. Adaptive Cruise Control (ACC) fills this gap and enables a convenient and safer driving experience by monitoring other vehicles and objects on the road with the use of sensors. It also helps the driver keep a steady vehicle speed.

The main aim of this manuscript is to prefer an applicable car with ACC together with its latest model by the Lattice Ordered Complex Linear Diophantine Fuzzy Soft Set algorithm.

Let {l_1_,l_2_,l_3_,l_4_} symbolize the Car with ACC. A balanced strategy to satisfy customer demands and industry requirements is ensured by giving careful consideration to cost for affordability, performance for efficiency, and sensor kinds for safety and technology integration. So let {h_1_,h_2_,h_3_} indicates the parameter set, where h_1_ = Cost, h_2_ = Performance and h_3_ = Sensor type used.

The fundamental aim of this manuscript is to prefer an applicable Car with ACC together with its latest model based on the expertise of the three experts namely {M, I, D}, where M is an Automotive Engineer for evaluating technical specifications, structural design, and overall vehicle functionality, I is an Environmental Analysts for emphasising on sustainability of the environment, evaluating the environmental effect of cars, and examining methods for implementing greener practices and technologies, and D is a Safety and Compliance Analysts for examining crash test outcomes, safety standards, and regulatory compliance to provide information on the level of security of a car. The hierarchy of the parameters is h_1_ ≤ h_2_ ≤ h_3_. The methodological steps of the proposed algorithm are illustrated below.

**Methodological steps:**

**Step 1:** Formulation of Lattice Ordered Complex Linear Diophantine Fuzzy Soft Matrix(LOCLDFSM) from the attribute values.

**Step 2:** Estimation of Score Matrix by using the formula.

S(M) = 1/ 4 (($\Gamma_{h_{pi}}^{M}$ −$\Delta_{h_{pi}}^{M}$) + ($w_{\Gamma_{h_{pi}}}^{M}$- $w_{\Delta_{h_{pi}}}^{M}$) + ($\alpha_{h_{pi}}^{M}$ −$\beta_{h_{pi}}^{M}$) + ($w_{\alpha_{h_{pi}}}^{M}$- $w_{\beta_{h_{pi}}}^{M}$ )), ∀ p=1,2,...,n and i=1,2,...,m, where M is a LOCLDFSM_n×m_.

**Step 3:** Construction of the Utility Matrix by using the formula.

U(M,I) = S(M) - S(I) where M and I are LOCLDFSM_n×m_.

**Step 4:** Formulation of Total Score Matrix.

**Step 5:** Ranking the alternatives.
